# Bile acids in immunity: Bidirectional mediators between the host and the microbiota

**DOI:** 10.3389/fimmu.2022.949033

**Published:** 2022-08-16

**Authors:** Urszula Godlewska, Edyta Bulanda, Tomasz P. Wypych

**Affiliations:** Laboratory of Host-Microbiota Interactions, Nencki Institute of Experimental Biology, Polish Academy of Sciences, Warsaw, Poland

**Keywords:** bile acids, microbial metabolites, immunity, intestinal inflammation, host-microbiota interactions

## Abstract

Host-microbiota interactions are bidirectional. On one hand, ecological pressures exerted by the host shape the composition and function of the microbiota. On the other, resident microbes trigger multiple pathways that influence the immunity of the host. Bile acids participate in both parts of this interplay. As host-derived compounds, they display bacteriostatic properties and affect the survival and growth of the members of the microbial community. As microbiota-modified metabolites, they further influence the microbiota composition and, in parallel, modulate the immunity of the host. Here, we provide a comprehensive overview of the mechanisms behind this unique dialogue and discuss how we can harness bile acids to treat intestinal inflammation.

## Introduction

Microbial communities residing in the gastrointestinal tract, collectively known as the gut microbiota, evolved various ways to interact with the immune system of the host (1). One such mechanism involves the production of endogenous metabolites or modification of host-derived products. Bile acids (BAs) constitute an important class of host-derived molecules that gain novel features *via* microbial transformation. Cholic (CA) and chenodeoxycholic acids (CDCA) in humans, and CA, CDCA, muricholic acids (αMCA and βMCA), and ursodeoxycholic acid (UDCA) in rodents, are synthesized from cholesterol in the liver and further metabolized to glycine- or taurine- conjugated bile acids (2, 3). Subsequently, conjugated bile acids are delivered to the gallbladder for storage, and after food intake, they are released into the small intestine to facilitate lipid digestion. Most BAs (up to 95%) are reabsorbed back to the liver and only a small portion that remains in the intestine can be further transformed by the gut microbiota into the secondary bile acids (4). The major microbial transformations of primary bile acids include i) deconjugation, ii) oxidation and epimerization of the 3-, 7-, and 12-hydroxyl groups, iii) 7-dehydroxylation, iv) esterification and v) desulfation (3). The main gut bacterial genera associated with these conversions include *Bacteroides*, *Clostridium*, *Lactobacillus*, *Bifidobacterium*, *Enterococcus*, and *Listeria* in BA deconjugation; *Bacteroides*, *Blautia*, *Clostridium*, *Eubacterium*, *Eggerthella*, *Roseburia* and *Ruminococcus* in the oxidation and epimerization; *Clostridium* and *Eubacterium* in 7-dehydroxylation; *Bacteroides*, *Eubacterium* and *Lactobacillus* in esterification; and *Clostridium*, *Fusobacterium*, *Peptococcus* and *Pseudomonas* in desulfation (3, 5–7). All these bacterial transformations shape the signaling properties of secondary bile acids, and as such, contribute to regulating mucosal physiology in health and disease (6).

## Utilization of bile acids by the microbiota and its downstream effects

Consisting of hydrophobic backbone and hydrophilic hydroxyl groups that form amphipathic structures, bile acids (BAs) resemble antimicrobial peptides (AMPs) and thus, have long been considered to restrict bacterial growth (8–10). The hydrophobicity of the backbone increases, while the number of hydroxyl groups decreases the antimicrobial potential of BAs (9). Because the gut lumen contains micromolar concentrations of chemically diverse bile acid pool, commensal microbes developed various ways to resist BA toxicity (11, 12). One such mechanism is enzymatic detoxification of BAs, which has a series of consequences. First and foremost, it directly improves the survival of species equipped with such detoxification machinery. For example, the activity of bile-salt hydrolases, which catalyze deconjugation of bile salts, improves bacterial survival during the bile challenge (12). Second, BA detoxification changes the luminal pool of BAs and thus, indirectly affects other members of the microbial community. For example, the ability of *Eggerthella lenta* to convert deoxycholic acid (DCA) to a less bacteriostatic isodeoxycholic acid (isoDCA) favors the growth of *Bacteroides ovatus* (9). Different susceptibility to toxic effects of bile acids and their “detoxification” products by the members of the microbiota translates into a detoxification chain of reactions, performed by multiple species. For example, to yield isoallolithocholic acid (isoallo LCA) from CDCA *in vitro*, a culture of three different bacterial species (*Clostridium scindens*, *E. lenta*, and *Parabacteroides merdae*) was required, while monocultures were insufficient (13). Another mechanism employed by certain microbes, such as lactobacilli and bifidobacteria, to survive in the presence of bile acids is their sequestration inside the cytoplasm (14). Its consequence for the whole microbial community can be the reduction of BA content, as exemplified by select *Lactobacillus* and *Bifidobacterium* strains, which were able to lower the concentration of deoxycholic acid in culture media (14, 15). Some bacteria take advantage of the differential sensitivity of microbes to bile acids to engage in competitive interactions. For example, *C. scindens* regulates the growth of *Clostridium difficile* by producing LCA and DCA, which are not only capable of inhibiting the growth of *C. difficile* directly (16), but also synergize with tryptophan-derived antimicrobials (17). This phenomenon was shown to be relevant *in vivo* since *C. scindens* or a bacterial consortium capable of producing pronounced amounts of LCA and DCA protected mice against *C. difficile* infection (16). This observation is consistent with the outgrowth of *C. difficile* after the selection of antibiotic regimens that efficiently depleted secondary BA-producing bacteria (18). Importantly, however, BA’s impact on virulence or colonization does not have to be unidirectional. For instance, lithocholic acid induced a morphotype switch in vancomycin-resistant *Enterococcus faecium* and promoted biofilm formation and intestinal colonization (19).

The impact of host/microbe-derived BAs on the microbiota composition might be most dramatic early in life when the microbiota is still in its immature stage. Post-weaning changes in concentrations of specific BA species correlated with dynamic changes in the relative abundance of certain bacterial taxa. Ursodeoxycholic acid (UDCA), glycine-conjugated cholic acid, taurine-conjugated α/β-muricholic acid (TMCA), and taurine-conjugated cholic acid (TCA) had the largest impact and predominantly determined the relative abundance of *Mannheimia*, *Streptococcus*, *Enterorhabdus* and *Lactobacillus* (20). When orally administered into neonatal mice, UDCA and βTMCA decreased the abundance of *Escherichia* and enhanced the abundance of *Lactobacillus*. In addition, βTMCA and TCA increased the richness of the small intestinal microbiota composition (20). These effects could be partially attributed to direct (albeit different) effects of these bile acids since UDCA inhibited the growth of *E.coli* while βTMCA promoted the growth of representative Lactobacilli isolates *in vitro*. While the precise mechanisms driving changes in the microbiota composition/richness *in vivo* remain elusive, two major scenarios are likely to occur in parallel: i) BAs shape the microbiota composition directly due to their antimicrobial properties (as discussed earlier), and ii) BAs promote the growth of certain microbes indirectly by inhibiting immune responses tailored to restrict their growth (the anti-inflammatory properties of BAs are described in the next section). The outcomes of both scenarios are subject to further forces from the progressively changing bile acid pool, other signals from transiently formed microbiota, and the maturing immune system. These pathways are not only bidirectional (BA pool – microbiota; immune responses – microbiota) but also intertwine with each other, creating a complex, multidimensional network that is actively engaged in the development of the mature intestinal ecosystem. Elements that are key to establishing healthy mucosal homeostasis are incompletely understood. Their identification might be pivotal to designing treatments or dietary supplements in a period in life that sets the individual on the trajectory toward health or disease.

**Figure 1:**
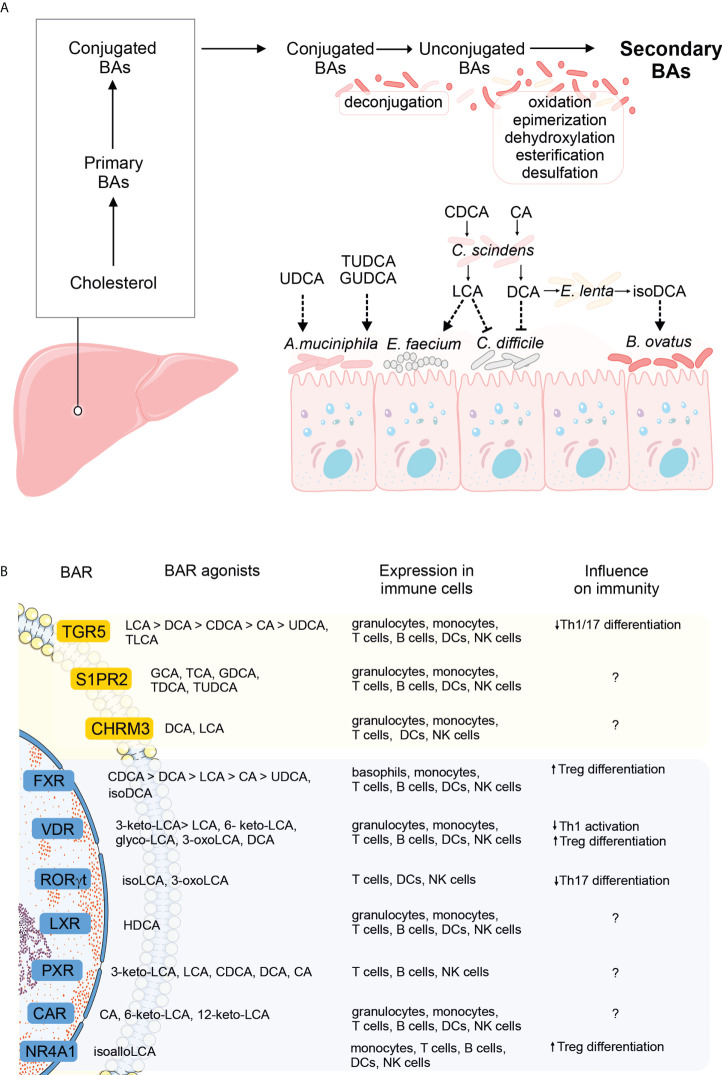
The influence of bile acid (BA) metabolism on the gut microbiome and host immunity. **(A)** Primary BAs are produced from cholesterol in the liver, conjugated to glycine or taurine, and secreted into the gut lumen. In the intestine, primary BAs undergo un-conjugation by the microbiota, followed by further rounds of modifications (oxidation and epimerization, dehydroxylation, esterification, or desulfation), to yield secondary BAs. Conjugated, unconjugated, and secondary BAs can all shape the microbial composition in the intestine (dashed arrows). Specific examples include unconjugated and taurine or glycine-conjugated UDCA promoting the growth of *A municiphila*, LCA derived from the conversion of CDCA by *C scindens* and promoting the growth of *E faecium* while inhibiting the growth of *C difficile*, DCA, derived from the conversion of CA by *C scindens* and inhibiting the growth of *C difficile* and finally, isoDCA, derived from the conversion of DCA by *E lenta*, and promoting the growth of *Bacteroides* (see also main text). **(B)** Schematic overview of the G-protein coupled (yellow) and nuclear receptors (blue) specific for bile acids (greater-than signs denote higher affinities). The panel of BARs expressed in immune cells was extracted from the Human Protein Atlas, using the HPA (26) and Monaco datasets (27). Immunomodulatory effects of receptor signaling by at least one natural ligand is noted (question marks denote receptor signaling with the influence on immunity that remains to be identified).

Finally, given bidirectional interactions between host/microbe-derived bile acids and the microbiome, it is not surprising that the bile acid pool is altered in dysbiosis. For example, patients suffering from inflammatory bowel disease (IBD) were reported to have decreased concentrations of secondary BAs in feces and serum (21, 22). Given the above, it is tempting to speculate if BA supplementation could improve dysbiosis in IBD patients. In mice, BA treatment (with UDCA and its glycine or taurine- conjugated species) prevented dextran sulfate sodium-mediated dysbiosis and promoted the growth of *Akkermansia muciniphila* [relative abundance of which is decreased in IBD patients (23, 24)], which provided proof-of-concept evidence that BAs can shape the microbiota composition (25). On the final note, the differences in BA pool in disease may not only be quantitative but also qualitative. For example, IBD patients had impaired desulfation of BAs, which resulted in increased levels of 3-OH-sulphated BA metabolites that lack anti-inflammatory potential (21, 22). Collectively, bile acids, first produced by the host, and later modified by the microbiota, constitute a unique class of compounds active along the host-to-microbe, and microbe-to microbe axes, that ultimately shapes the microbiota composition of the host (**Figure 1A**, **Table 1**).

**Table 1:** The role of bile acids in shaping the microbiota composition.

Bile acid	Effect on the microbiome (*in vivo*)	Mechanism (*in vitro*)	Reference
**Primary BAs**	UDCA decreases the abundance of *Escherichia* and enhances the abundance of *Lactobacillus* when administered to neonatal mice	Directly inhibits the growth of *Escherichia*	(20)
	UDCA promotes the growth of *Bacteroidaceae*, *Clostridium* cluster XIVa and *Akkermansia* during the DSS model of colitis	Unknown	(25)
**Conjugated BAs**	βTMCA decreases the abundance of *Escherichia* and enhances the abundance of *Lactobacillus* when administered to neonatal mice; βTMCA and TCA increase microbiota richness when administered to neonatal mice	Directly promotes the growth of *L. johnsonii* and *L. reuteri*	(20)
	glycine-conjugated UDCA (GUDCA) promotes the growth of *Bacteroidaceae*, and *A. muciniphila* during the DSS model of colitis; taurine-conjugated UDCA (TUDCA) promotes the growth of *Prevotellaceae* and *A. muciniphila* during the DSS model of colitis	Unknown	(25)
**Secondary BAs**	Adoptive transfer of DCA and LCA-producing bacterial consortium or *C. scindens* enhances resistance to *C.difficile* infection	DCA and LCA each inhibit the growth of *C. difficile* directly; DCA and LCA enhance the activity of antimicrobials produced by *C. scindens*	(16), (17)
	Antibiotic treatment targeting secondary bile acid-producing bacteria enhances the outgrowth of *C. difficile*	LCA, DCA and HDCA (hyodeoxycholic acid) directly inhibit the growth of *C. difficile*	(18)
	–	Conversion of DCA by *E. lenta* to a less bacteriostatic isoDCA favors the growth of *Bacteroides ovatus*	(9)
	LCA promotes biofilm formation and intestinal colonization of vancomycin-resistant *E. faecium*	LCA induces MgCl_2_-dependent morphotype switch to chained growth	(19)

## The influence of secondary bile acids on immunity

Technological advances in next-generation sequencing and the development of bioinformatics tools for data analyses in the last two decades opened the door to our understanding of how profoundly the microbiota shapes immunity. This sparked an interest in delineating microbial species and their products that would exert immunomodulatory effects, and defining signaling pathways they trigger. Given the above, when bile acid receptors were identified, researchers sought to investigate if microbiota-dependent modifications of primary bile acids might be important from the immunological standpoint. Indeed, secondary bile acids were shown to modulate innate immunity and influence the severity of experimentally-induced diseases. For example, intraperitoneal administration of tauroursodeoxycholic acid in mice reduced the expression of antigen presentation machinery in the gut, prevented apoptosis of intestinal epithelial cells, and improved the outcome of intestinal acute graft-versus-host disease (28). Deoxycholic acid (DCA), but not its precursor, cholic acid (CA), modulated the function of dendritic cells (DCs) and protected mice against experimental autoimmune uveitis. Mechanistically, DCs from mice fed a DCA-enriched diet, but not a CA-enriched diet, were less responsive to LPS priming *in vitro*, and DCA pretreatment reduced surface expression of co-stimulatory molecules and MHC-II, and led to a less pronounced differentiation of Th1/17 subsets *in vitro* (29). The immunomodulatory effects of bile acids are initiated by their interactions with bile acid receptors, expressed in various immune cell types, including monocytes, T cells, B cells, DCs, NK cells, and granulocytes (details included in **Figure 1B**). Notably, many bile acid receptors bind several ligands with different affinities (**Figure 1B**). For instance, TGR5 is bound by bile acids with affinities descending in the following order: LCA>DCA>CDCA>CA>UDCA, TLCA (6), while FXR by acids in the following order: CDCA>DCA>LCA>CA>UDCA, isoDCA (30). Fine-tuning BAR activation plays a critical role in dictating the immunological outcome. Intriguingly, the highest affinity does not necessarily guarantee the most pronounced effect. For example, FXR activation by isoDCA (low affinity) but not CDCA, DCA, LCA, CA, or UDCA (higher affinities) programmed DCs to induce differentiation of Treg cells *in vitro* (31). This example points to the complexity of BA-BAR interactions and highlights the risk of not recapitulating their effects by pharmacological activation with artificial BAR agonists (discussed further in the next section). Other examples of receptors that bind several bile acids include S1PR2 (bound by GCA, TCA, GDCA, TDCA, and TUDCA), VDR (bound by 3-keto-LCA> LCA, 6- keto-LCA, glyco-LCA, 3-oxoLCA, and DCA), PXR (3-keto-LCA, LCA, CDCA, DCA, CA), and CAR (CA, 6-keto-LCA, 12-keto-LCA) (**Figure 1B**). Detailed comparisons of the efficacy of each ligand in achieving the desired outcome in disease settings and defining the optimal affinity of this interaction are the challenge for further studies.

In addition to acting on innate immune cells, bile acids were also shown to directly modulate adaptive immunity. Lithocholic acid (LCA) inhibited activation of Th1 cells *in vitro via* Vitamin D Receptor (VDR) signaling (32). 3-oxolithocholic and isolithocholic acids inhibited Th17 differentiation *in vitro* and *in vivo* by binding RORγt, a master regulator of the Th17 subset, and inhibiting its transcriptional activity (33, 34). Isoallolithocholic acid (isoalloLCA) promoted the generation of mitochondrial reactive oxygen species, leading to enhanced expression of FOXP3 and differentiation of regulatory T cells *in vitro*. Interestingly, isoalloLCA was not sufficient to influence the Treg cell pool *in vivo*, but increased colonic Treg cell pool in combination with 3-oxoLCA. This was unexpected since 3-oxoLCA by itself did not affect the Treg cell pool *in vitro* or *in vivo*. The mechanisms of action behind this synergistic effect have not been delineated (33). The complexity behind the action of secondary bile acids and their combinations has been further demonstrated in a subsequent report where none of the bile acids (including LCA or 3-oxoLCA) was able to influence Th17 responses *in vivo*. When screening for the capacity to induce Treg cell differentiation, the authors concluded that the combination of 3-oxoLCA and LCA is capable of maintaining high frequencies of this cell subset *in vivo* and that it depended on VDR expression on Treg cells (35). Overall, these reports drew a general picture that lithocholic acid derivatives hold the potential to induce regulatory T cells, especially when used in combination. Differences in regards to specific LCA species identified in these reports and their mechanisms of action might stem from differences in experimental setups. In a study by Pols et al., a Jurkat T cell line was used (32), Hang et al., used a differentiation protocol based on cytokine stimulation of sorted naïve T cells (33) while Song et al., fed mice with diets enriched in single bile acids or their mixtures, and evaluated the frequency of colonic Treg cells *ex vivo* (35). Altogether, these examples point to the caution when designing the appropriate screening strategy and highlight the need to validate obtained results *in vivo*.

Despite these insights into the role of secondary bile acids in shaping T helper cell fate, its relevance in health and disease remains elusive. The influence of LCA, 3-oxoLCA, and 3-oxoLCA/isoalloLCA in disease settings has not been evaluated (32, 33), similarly as in the case of isoDCA (31). In a study by Song et al., the relevance of bile acid treatment in a mouse model of colitis was shown, whereby feeding mice with primary or secondary bile acids ameliorated the severity of this condition (35). The capacity of primary bile acids to confer this effect might reflect their conversion into secondary bile acids in mice; however, this possibility has not been confirmed with the use of germ-free mice. When it comes to the relevance of secondary BAs in human patients, the data is even scarcer. Nevertheless, one study pointed to the reduced levels of 3-oxoLCA/isoLCA in inflammatory bowel disease patients as well as to the reduced relative abundance of a bacterial gene involved in 3-oxoLCA/isoLCA biosynthesis, 3α-hydroxysteroid dehydrogenase (34).

On the final notes, it is worth acknowledging that although anti-inflammatory properties of secondary bile acids are generally perceived as beneficial, their potentially harmful role was also reported. Using mouse models of liver cancer, the authors linked the capacity of the antibiotic treatment to inhibit liver tumor growth with a reduction in secondary BA levels in the liver. Treating mice with LCA or ω-muricholic acid (ω-MCA) reversed the beneficial effect of the antibiotic treatment. Mechanistically, LCA or ω-MCA blunted the expression of CXCL16, a chemokine driving recruitment of natural killer T cells to the tumor site. This effect might be restricted to the liver since antibiotic treatment did not reduce tumor growth or metastasis of subcutaneous or lung tumors, respectively (36). Finally, it is worth noting that, although the anti-inflammatory mode of action behind secondary bile acids is well established, scarce reports exist on their pro-inflammatory potential. A cholic acid diet or a deoxycholic acid treatment reduced the frequency of tuft cells in the biliary tract. Interestingly, the abundance of tuft cells negatively correlated with a neutrophil influx in a model of experimental cholestasis, and tuft cell deficiency increased biliary neutrophilia under homeostatic conditions. This interplay was modulated by the microbiome since microbiota transfer between mice from different providers reversed vendor-dependent variation in the tuft cell/neutrophil ratios. These observations, though indirect, outline the possibility that secondary bile acids increase neutrophilia by reducing the frequency of biliary tuft cells (37). The up-to-date list of bile acid species reported to modulate inflammation and the summary of their mechanisms of action is presented in **Figure 1B** and **Table 2**.

**Table 2:** Immunomodulatory properties of secondary bile acids.

Bile acid	Receptor involved	Cellular mechanisms	Effect on disease	References
**Innate immunity**				
DCA	Unknown	Reduces frequency of tuft cells. Increases biliary neutrophilia	Might exacerbate obstructive cholestasis	(37)
DCA	TGR5	Reduces secretion of IL-1β, IL-6, IL-12p70, and TNF-α;	Protects mice against experimental autoimmune uveitis	(29)
TUDCA	Unknown	Reduces surface expression of co-stimulatory molecules (CD40, CD80 and CD86) and MHC-II; Reduces differentiation of Th1/17 Reduces the expression of antigen presentation machinery in the gut; Dampens innate inflammatory response to IFN-γ; Attenuates T-cell activation	Ameliorates intestinal aGvHD disease	(28)
isoDCA	FXR	Modulates dendritic cell function to induce Treg cells	Unknown	(31)
**Adaptive immunity**				
LCA	VDR	Inhibits Th1 cell activation *in vitro*	Unknown	(32)
3-oxoLCA	RORγt	Inhibits Th17 cell differentiation *in vitro* and *in vivo*	Unknown	(33)
isoLCA 3-oxoLCA /isoLCA	RORγt	Inhibits Th17 cell differentiation *in vitro* and *in vivo*	3-oxoLCA/isoLCA levels reduced in IBD patients	(34)
isoalloLCA or isoalloLCA/3-oxoLCA	Unknown	Enhances Treg cell differentiation *in vitro* or *in vivo*	Unknown	(33)
3-oxoLCA /LCA	VDR	Enhances Treg cell differentiation *in vivo*	Ameliorates colitis-induced inflammation in mice	(35)

## Therapeutic potential of bile acids

Immunomodulatory properties of secondary bile acids open up three major avenues that might be considered clinically in a fight against intestinal inflammation: 1) pharmacological activation of bile acid receptors, 2) direct application of secondary bile acids, 3) administration of microbes capable of producing them. The advantage of pharmacological activation of bile acid receptors is their specificity, since triggering defined immunological pathways limits possible side effects. Agonists of two bile acid receptors, TGR5 (GPBAR1) and FXR, have attracted the most attention as drug candidates, and their efficacy in preclinical models of colitis has been widely described (38–42). Despite this, no clinical trials targeted against intestinal inflammation have been launched so far (as of May 2022, according to clinicaltrials.gov). Conducting large-scale, randomized clinical trials will be pivotal to indicate the utility of bile acid receptor agonists to ameliorate intestinal inflammation.

An alternative approach to pharmacological activation of bile acid receptors is a direct application of desired secondary bile acid species or the bacteria equipped with machinery to produce them. The advantage of this approach is the possibility to fine-tune BAR activation (since the magnitude of BAR stimulation varies according to BA species) (43–45). This might be particularly important considering the role of optimal (not necessarily the highest) affinity in mediating the desired effect (as discussed in a previous section). Also, unlike in the case of pharmacological activation, secondary bile acids can trigger multiple BARs. For example, LCA activates FXR (43–45), VDR (46), TGR5 (47, 48), and PXR (49), while DCA activates FXR (43) and TGR5 (47, 48). The pleiotropic action of bile acids might be advantageous in the face of immunological redundancy that drives inflammation. The proof-of-concept experiments showing the health benefits of secondary bile acid administration in mouse models of intestinal inflammation have been discussed in a previous section (28, 35). Pilot trials concerning the use of microbes capable of producing them were only conducted in germ-free (GF) settings. Administration of wild-type *Bacteroides thetaiotaomicron* and *Bacteroides fragilis* strains capable of producing secondary bile acids, induced colonic RORγt^+^ Treg cells when administered into GF mice (35). Likewise, an isoDCA-producing consortium of bacteria, induced intestinal lamina propria RORγt^+^ Treg cell pool (31). Finally, administration of 3α-hydroxysteroid dehydrogenase-expressing strains of *E. lenta* and/or *B. fragilis* into GF mice fed with LCA led to increased production of 3-oxoLCA and isoLCA (34). Although encouraging, these conclusions should be confirmed in organisms with complex microbiomes since the administration of live bacteria to already inhabited ecological niches comes at the risk of unsuccessful colonization of the administered microbes, or alterations in their function. The consequence of that might be loss of efficacy in some individuals or the development of side effects. Given the variability of microbiota composition in humans, ascertaining clinical safety and efficacy in yielding desired bile acid species should be conducted on a large number of participants, whose microbiota composition is characterized. Such an approach might provide precise estimates of the success rate of the designed regimen and link it to microbial signatures of the responders, which would be critical for designing follow-up therapeutic strategies.

## Conclusions and perspectives

In summary, bile acids represent a single class of mediators with bidirectional effects in the host-microbiota dialogue. When host-derived, they shape the composition of the microbiota. When modified by the microbiota, they further shape the microbiota composition and in addition, they influence the immunity of the host. Each reaction constitutes a tiny piece of the complex network that leaves marks on the microbial and the mammalian parts of the ecosystem. Understanding the mechanisms that govern this interplay constitutes a major workload for basic research, as fundamental questions remain open. For example, which interactions within this network are crucial to maintaining immunological tolerance in the gut? How important is the timing of their occurrence following birth? When absent or delayed, can they actively contribute to disease development and can we reduce the symptoms by altering the bile acid pool? What strategy is most effective in preclinical settings (repeated administration of BAs, pharmacological activation of BARs or downstream signaling pathways, administration of microbes/microbial consortia equipped with the machinery to yield desired BA species, etc.)? Each of these questions requires a tremendous number of experiments from different scientific fields, including immunology, microbiology, chemistry, and medicine. Nevertheless, data obtained from these experiments will provide grounds for formulating applied questions and in the long run, might pave the way to targeting pathways of bile acid metabolism for human health benefits.

## Author contributions

UG, EB, and TW wrote the manuscript. UG prepared the figure, UG and EB prepared the tables. All authors critically read the manuscript and accepted the submitted version.

## Funding

TW is supported by grants from National Science Centre (SONATA 16, grant no. 2020/39/D/NZ6/02146 and OPUS 21, grant no. 2021/41/B/NZ6/02219).

## Acknowledgments

Parts of the figure presented in **Figure 1** were adapted from Servier Medical Art (http://smart.servier.com) and used under a Creative Commons Attribution 3.0 Unported License (CC BY 3.0).

## Conflict of interest

The authors declare that the research was conducted in the absence of any commercial or financial relationships that could be construed as a potential conflict of interest.

## Publisher’s note

All claims expressed in this article are solely those of the authors and do not necessarily represent those of their affiliated organizations, or those of the publisher, the editors and the reviewers. Any product that may be evaluated in this article, or claim that may be made by its manufacturer, is not guaranteed or endorsed by the publisher.
